# Fig-YOLO: An Improved YOLOv11-Based Fig Detection Algorithm for Complex Environments

**DOI:** 10.3390/foods14234154

**Published:** 2025-12-03

**Authors:** Zhihao Liang, Ruoyu Di, Fei Tan, Jinbang Zhang, Weiping Yan, Li Zhang, Wei Xu, Pan Gao, Zhewen Hao

**Affiliations:** 1 College of Information Science and Technology, Shihezi University, Shihezi 832003, China; liangzhihao@stu.shzu.edu.cn (Z.L.); diruoyu@stu.shzu.edu.cn (R.D.); tfnszbd@163.com (F.T.); zhangjinbang@stu.shzu.edu.cn (J.Z.); yanweiping@stu.shzu.edu.cn (W.Y.); zl_inf@shzu.edu.cn (L.Z.); 2 Key Laboratory of Physiological and Quality Control of Specialty Fruits and Vegetables, College of Agriculture, Shihezi University, Shihezi 832003, China; xuwei0412@shzu.edu.cn

**Keywords:** fig detection, Fig-YOLO, YOLOv11n, complex environments

## Abstract

Accurate fig detection in complex environments is a significant challenge. Small targets, occlusion, and similar backgrounds are considered the main obstacles in intelligent harvesting. To address this, this study proposes Fig-YOLO, an improved YOLOv11n-based detection algorithm with multiple targeted architectural innovations. First, a Spatial–Frequency Selective Convolution (SFSConv) module is introduced into the backbone to replace conventional convolution, enabling joint modeling of spatial structures and frequency-domain texture features for more effective discrimination of figs from visually similar backgrounds. Second, an enhanced bi-branch attention mechanism (EBAM) is incorporated at the network’s terminal stage to strengthen the representation of key regions and improve robustness under severe occlusion. Third, a multi-branch dynamic sampling convolution (MFCV) module replaces the original C3k2 structure in the feature fusion stage, capturing figs of varying sizes through dynamic sampling and residual deep-feature fusion. Experimental results show that Fig-YOLO achieves precision, recall, and mAP@0.5 of 89.2%, 78.4%, and 87.3%, respectively, substantially outperforming the baseline YOLOv11n. Further evaluation confirms that the model maintains stable performance across varying fruit sizes, occlusion levels, lighting conditions, and data sources. Fig-YOLO’s innovations offer solid support for intelligent orchard monitoring and harvesting.

## 1. Introduction

*Ficus carica* L., commonly known as fig, is a long-established subtropical deciduous fruit tree widely distributed across the world, particularly in the Mediterranean region, where it holds significant economic value [1,2,3,4]. In China, Atush City in Xinjiang is renowned for its high-quality figs with unique flavors and is often referred to as the “Home of Chinese Figs.” Xinjiang figs are a regionally significant specialty product, and the sustainable development of fig cultivation is crucial for improving the incomes of local residents. However, fig harvesting still largely depends on manual labor, which is not only inefficient and labor-intensive but also increases harvesting costs and operational risks [5]. In the field of agricultural automation, the application of machine vision recognition systems in harvesting robots to achieve accurate fruit identification in complex environments is a current research focus and challenge [6]. This issue is particularly evident in figs. Figs are small in size, and their pedicels are closely attached to the branches, making the fruits prone to unnecessary damage during manual harvesting. Furthermore, figs grow in environments with dense foliage, and their fruit color closely resembles the background, which further complicates precise identification. Therefore, improving the detection accuracy of figs is of paramount importance.

In traditional image processing workflows, fruit images typically require separation from the background using methods such as threshold segmentation or K-means clustering, followed by the extraction of handcrafted features. These features are then combined with classifiers such as Artificial Neural Networks (ANNs), Support Vector Machines (SVM), Convolutional Neural Networks (CNNs), or Random Forests (RFs) for fruit recognition and classification [7]. Thendral et al. [8] proposed two segmentation methods, edge-based and color-based, for segmenting orange fruit images under natural lighting conditions. The study used 20 randomly selected digital orange images from the internet for experiments, and the results showed that the color-based segmentation method outperformed the edge-based method in terms of localization accuracy and segmentation effectiveness. Kuang et al. [9] proposed a multi-class fruit detection method based on multiple color channels. By extracting color histograms, LBP, HOG, and GaborLBP features, and selecting the optimal feature blocks for multi-feature fusion, this method produced a false positive rate of 0.5427 FPPI with a missed detection rate of 0.156, achieving a high average accuracy of 0.8135. This method effectively handled fruit detection under different backgrounds, positions, angles, and sizes. Ratnasari [10] proposed a fruit recognition and classification method by constructing color and texture features of fruit images, combined with a feature co-occurrence-based K-Nearest Neighbors (K-NN) algorithm. Experiments on a dataset of 12 categories with a total of 1882 fruit images showed a maximum classification accuracy of 92%. Rachmawati et al. [11] proposed a Hierarchical Multi-Feature Classification (HMC) method for multi-class fruit recognition. This method combined color features with RGBD 3D shape features and utilized the hierarchical structure of fruit species and varieties for classification, significantly improving classification performance. Nosseir et al. [12] introduced a fruit recognition method based on Gray-Level Co-occurrence Matrix (GLCM) color and texture features, employing multiple K-NN classifiers for classification. When evaluated with 46 seasonal fruit images (strawberry, apple, banana), the classifier’s accuracy reached up to 96.3%, and the overall recognition rate reached 100%. However, the above traditional image processing methods depend on color, shape, and texture features in the images, making them less effective in handling complex environmental conditions such as lighting changes, complex backgrounds, and fruit occlusions. Their ability requires extensive human intervention, making large-scale automation applications difficult.

In contrast, deep learning has shown significant advantages in fruit detection and automatic harvesting tasks. Biswas et al. [13] proposed a fruit recognition and classification method based on Deep Convolutional Neural Networks (CNNs). By optimizing the parameters, they applied CNN for multi-class fruit detection and recognition. In experiments with 1200 images of apples, lemons, tomatoes, and plums, the classification accuracy was 98%, significantly outperforming traditional methods like SVM. Min et al. [14] introduced a Multi-Scale Attention Network (MSANet) for fruit recognition. By extracting attention features from different layers of the CNN and aggregating multi-layer visual attention into a comprehensive representation, experiments on four fruit benchmark datasets showed that MSANet outperformed baseline CNN methods in recognition performance. He et al. [15] proposed a tomato detection model, T-Net, based on YOLOv3, to capture the 3D information of mature tomatoes in greenhouses. The model used the ROS environment to obtain tomato posture via the visual coordinate system, enabling tomato recognition and localization. Yi et al. [16] proposed a fruit detection method based on an improved YOLOv4, incorporating SE attention mechanisms in the CSPResblock module, optimizing the SPP module, and refining the PANet structure. Experiments with a dataset of 8000 images from eight fruit types showed an average precision (mAP) of 98.02% and an accuracy of 95.62%. Chen et al. [17] proposed a multi-class occlusion target detection method, YOLO-COF, for Camellia oleifera fruit. The K-means++ clustering algorithm was introduced under the YOLOv5s framework to automatically filter the target dataset, and the Coordinate Attention module was incorporated to enhance the feature extraction capability for occluded targets. Shi et al. [18] introduced the YOLOv7-SAP model for young apple fruit detection in natural environments to improve bagging robot detection efficiency. This method incorporated SE attention blocks in the YOLOv7 backbone network and added the ASPP module at the front to enhance feature extraction for young fruits, achieving a mAP of 91.2% when young fruit color closely resembled the background. Su et al. [19] proposed the YOLOv8n-Pose-DSW model for zucchini recognition and harvesting point localization under challenging conditions in greenhouses, such as leaf occlusion, high density, and complex structures. This method replaced traditional upsampling operators with Dysample adaptive point sampling and used the WIoU-v3 loss to optimize bounding box regression, achieving effective fruit detection and harvesting point localization in complex environments.

These research methods have made significant progress in fruit detection. However, compared to detecting larger fruits like tomatoes or apples in greenhouse and natural environments, the recognition of figs in complex environments still faces many challenges. First, the target scale of figs is generally smaller, which makes them more likely to be missed during detection. Second, figs grow in clusters, with significant overlap between fruits, complicating precise segmentation and recognition. Intertwined branches and leaves often obscure the fruits, sometimes making them partially or entirely invisible, further increasing the detection difficulty. Third, the maturation process of figs is asynchronous, and the color of the fruits changes from green to yellow or green–yellow, which closely resembles the leaves both in color and texture, leading to misidentification. Finally, lighting variations in natural environments also affect recognition performance.

Kamaruzaman et al. [20] conducted a systematic literature review (SLR) on the application of deep learning in fig fruit detection and counting, employing the ROSES assessment framework to perform structured screening and analysis of relevant studies. Their review ultimately selected 33 representative works related to fruits and vegetables from the IEEE, Scopus, and Web of Science databases, and indicated that various deep learning algorithms are capable of achieving effective fig fruit recognition and counting in field environments. Yijing et al. [21] proposed a fig fruit recognition method based on YOLOv4, achieving high precision (Precision = 95.03%) on a public dataset, with an inference time of approximately 117.4 ms per image, enabling effective fruit localization. However, the method did not investigate the applicability of the model under complex environmental conditions, relied on a limited set of evaluation metrics, and exhibited relatively long inference time. Kamaruzaman et al. [22] proposed a deep learning-based approach for fig fruit detection. Images were collected under natural growth conditions and manually annotated with the label “buah tin” to construct a dataset. Using Python on the Google Colaboratory platform, the authors trained and evaluated both YOLOv3 and YOLOv4 models. Experimental results demonstrated that YOLOv4 outperformed YOLOv3 in terms of mean average precision (mAP) and overall detection performance, achieving a maximum mAP of 90.02%, while the precision reached only 80%. Furthermore, the dataset constructed in this study covered relatively limited scenarios.

To address the above issues, we propose the Fig-YOLO model. Existing YOLOv11 enhancement studies primarily focus on improving detection accuracy or inference speed in general scenarios, yet they still exhibit limitations in feature extraction when applied to specific agricultural environments. To address the challenges commonly observed in complex fig orchard scenes, Fig-YOLO introduces dedicated feature enhancement structures to improve detection accuracy and stability in crop-specific settings, and conducts targeted evaluations in representative scenarios. Therefore, the innovation of Fig-YOLO lies in task-driven, scenario-oriented optimization rather than routine performance tuning of YOLOv11. In addition, Fig-YOLO not only improves detection accuracy but also provides applications with practical agronomic significance. Accurate fruit localization enables the estimation of fruit load and spatial distribution, which are essential for yield prediction, field-scale growth monitoring, and harvesting route planning. Enhanced boundary recognition also improves the reliability of subsequent phenotypic measurements, such as fruit maturity assessment, thereby supporting precision orchard management. By integrating visual detection results with agronomic variables, Fig-YOLO offers actionable insights for practical production beyond a purely technical case study. The specific objectives are as follows:

1. We establish a fig dataset collected in orchard environments with complex backgrounds, providing richly annotated samples for fig recognition research.
2. A SFSConv module is introduced, which simultaneously models local spatial structure and frequency domain texture features, thereby improving fruit boundary segmentation accuracy and enhancing the ability to distinguish against texture-similar backgrounds.
3. A dual-branch attention mechanism, EBAM, is proposed, which highlights key features with low computational overhead and reduces interference caused by occlusion in fig detection.
4. An MFCV module is introduced to efficiently fuse shallow detail information with deep semantic information, enhancing the model’s feature representation ability and robustness.

## 2. Materials and Methods

**Figure 1:**
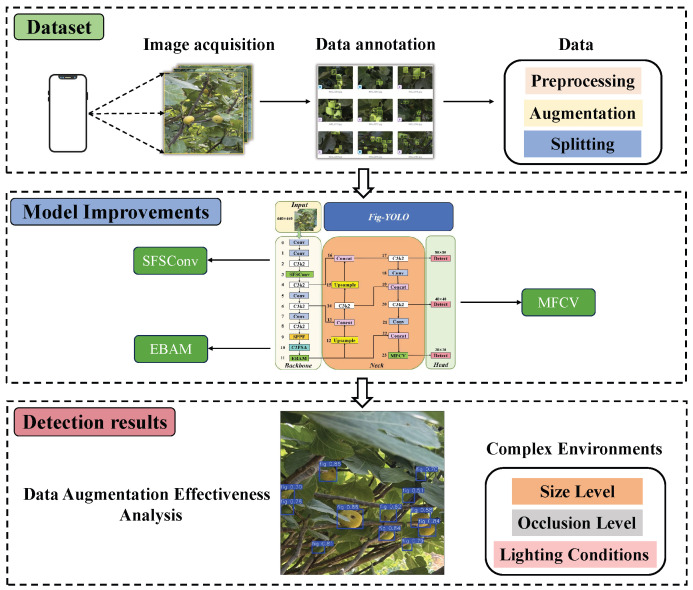
Overall workflow of this study.

This study mainly consists of three parts: dataset construction, model improvement, and detection result analysis. The overall workflow is shown in Figure 1.

### 2.1. Dataset Construction

The experimental data were collected from the Jinjiamu Orchard (39.70° N, 76.15° E) in Atush City and the Azihan Fig Ecological Garden (39.71° N, 76.17° E) in the Kizilsu Kirgiz Autonomous Prefecture of Xinjiang, as shown in Table 1. These locations are managed orchard environments, representing typical commercial fig production settings with controlled cultivation practices. Detecting figs in such orchards is of practical significance, as it enables accurate quantification of fruit load at the individual tree or orchard level, providing foundational data for yield prediction.

**Figure 2:**
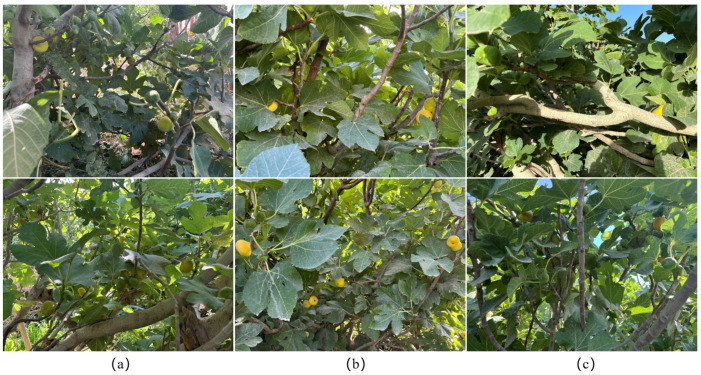
Representative fig fruit images: (**a**) small objects; (**b**) occlusion; (**c**) background similarity.

**Figure 3:**
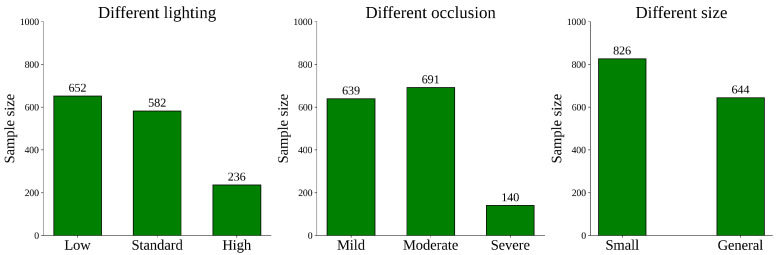
Visualization of label distribution in the dataset.

The fig variety used was the Xinjiang Early Yellow. The identification and verification of fig fruits were performed by trained orchard staff to ensure annotation accuracy, while non-fruit parts that were not collected are not included in the dataset. The images focus on individual fruits rather than branches or entire trees, providing clear examples for accurate fruit recognition. Data collection was conducted in early October 2024 and mid-July 2025, yielding a total of 1015 raw images in 2024 and 1861 raw images in 2025. During the data preprocessing stage, all images were carefully screened, and those that were blurry, overexposed, or exhibited obvious compression artifacts were removed. 950 and 1470 usable images were obtained at the Fig Ecological Garden and the Jinjiamu Orchard, respectively, resulting in a total of 26,315 fig samples. Through this procedure, we minimized the impact of compression artifacts inherent in high-resolution JPG images and ensured the clarity and usability of the training data. The collection process took into account diverse real production scenarios, as illustrated in Figure 2. The dataset distribution is shown in Figure 3.

The imaging devices used were a Redmi Note 12 Turbo (Xiaomi Corporation, Beijing, China) and an iPhone 15 Plus (Apple Inc., Cupertino, CA, USA), with resolutions of 3472 × 4624 and 4284 × 5712 pixels, respectively. All images were saved in JPG format for subsequent data processing and analysis. Images were captured at two times of the day, in the morning around 10:00 and in the afternoon around 16:00, to include different lighting conditions. The images were taken from a distance of approximately 0.5–1.5 m from the fruit, providing a clear view of individual figs while capturing the surrounding context. The data from both years were annotated using the labeling tool Roboflow, and the annotations were stored in YOLO TXT format. The 2025 dataset, which was larger, was used for modeling and randomly split into training, validation, and test sets in a 7:2:1 ratio, resulting in 1029 images for training, 294 for validation, and 147 for testing. The 2024 data serves as a supplementary test set for feasibility testing in complex environments.

Image preprocessing involved two main steps: Resize (640 × 640) and Tile (2 × 2). To increase data diversity and mitigate the risk of model overfitting, data augmentation techniques were applied to the images, expanding the training and validation sets to 12,348 and 1172 images, respectively.

### 2.2. Model Improvement and Training

#### 2.2.1. YOLOv11

Since its inception, the YOLO (You Only Look Once) family of models has made significant progress in real-time object detection. YOLOv11, released by Ultralytics in September 2024, introduces five model variants—YOLOv11n, YOLOv11s, YOLOv11m, YOLOv11l, and YOLOv11x [23]—supporting object detection, instance segmentation, and pose estimation tasks in computer vision.

**Figure 4:**
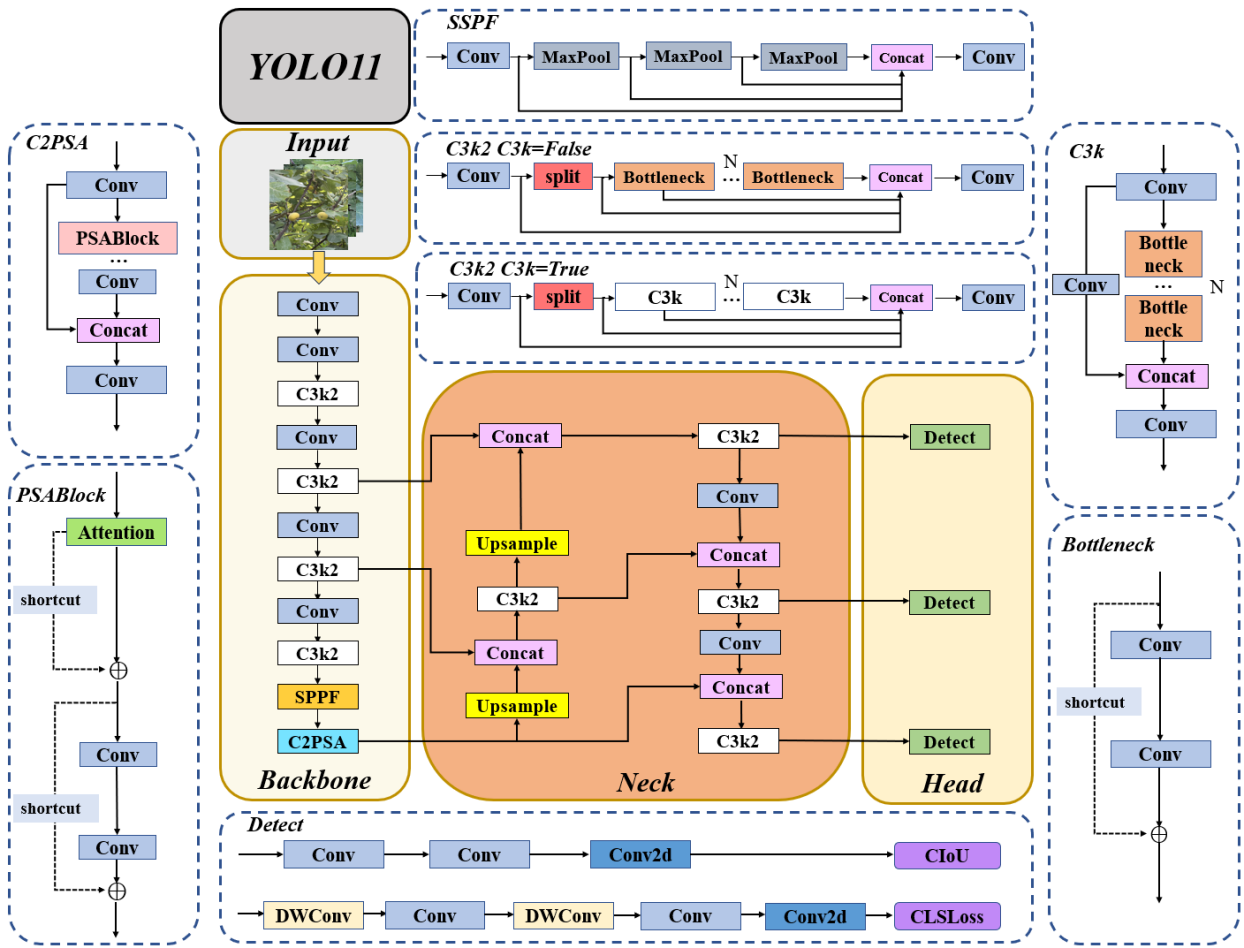
Overall network architecture of YOLOv11. “⊕” denotes residual connection.

YOLOv11 improves feature extraction while maintaining computational efficiency by introducing the enhanced C3k2 block, SPPF module, and C2PSA component, as illustrated in Figure 4. The C3k2 block optimizes convolutional kernel design to improve feature representation, while the SPPF module expands the receptive field and extracts multi-scale contextual information without increasing inference latency. The C2PSA component incorporates a parallel spatial attention mechanism to strengthen key feature extraction [24]. Additionally, the Neck of YOLOv11 aggregates features from different stages using C3k2 blocks, upsampling, and concatenation operations. This process ensures effective multi-scale feature fusion, enabling the network to preserve high-level semantic information while maintaining sufficient spatial resolution for small object detection. The Head, composed of the Detect module, performs convolutional operations on the fused features to predict object locations and confidence scores. During training, the loss functions—including CIoU loss for bounding box regression and classification loss for object categorization—guide the network to optimize both localization and classification simultaneously. Further details are available in the official YOLOv11 documentation by Ultralytics [25].

#### 2.2.2. Improved YOLOv11 Fig Detection Algorithm

**Figure 5:**
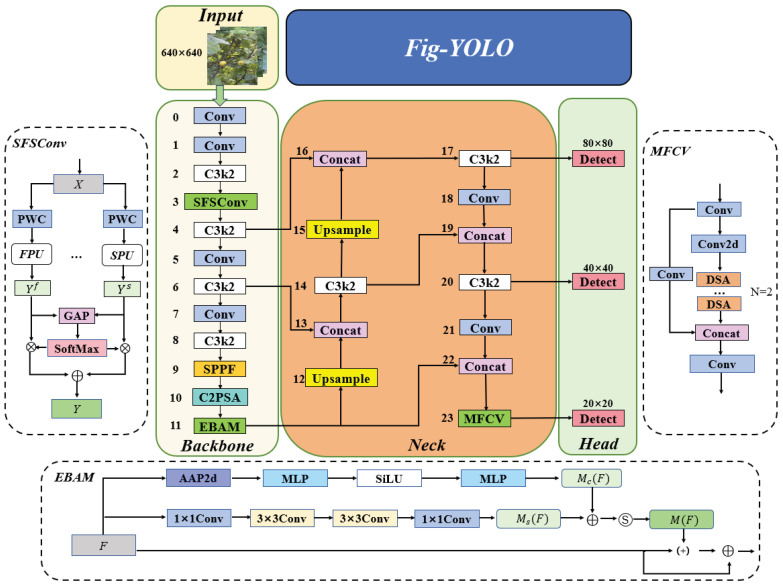
Network architecture of the proposed Fig-YOLO model. The dashed boxes indicate the workflow of the three introduced modules.

To achieve efficient fig detection in complex environments, we propose a new model, Fig-YOLO, based on the YOLOv11n architecture, as shown in Figure 5. The solid-line structure represents the basic backbone–neck–head architecture of YOLOv11n, and the dashed boxes specifically depict the three newly introduced custom modules (SFSConv, EBAM, MFCV) and their internal workflows.

In the backbone network, the original third convolutional module is replaced with the SFSConv block, which enables simultaneous modeling of spatial and frequency-domain features in shallow layers. This enhances the model’s ability to differentiate figs from complex backgrounds and produces more discriminative features for multi-scale fusion. At the end of the backbone, we introduce an improved attention module, EBAM, which uses dual-branch channel and spatial attention to highlight key features with minimal computational cost. This design allows the model to maintain high detection accuracy even under occlusion by branches and leaves or when fruits overlap.

In the neck, only the C3k2 module in the 23rd layer is replaced with the MFCV module. This improves the perceptual and adaptive alignment of input features to the detection head, enhancing small-object detection accuracy while avoiding gradient instability and excessive computation that might arise if all layers were replaced.

Finally, the detection head retains three scale outputs (P3, P4, and P5) to detect small, medium, and large objects. Fig-YOLO, through the integrated design of SFSConv, EBAM, and MFCV, achieves complementary feature enhancement and significant improvement in accuracy.

#### 2.2.3. SFSConv

**Figure 6:**
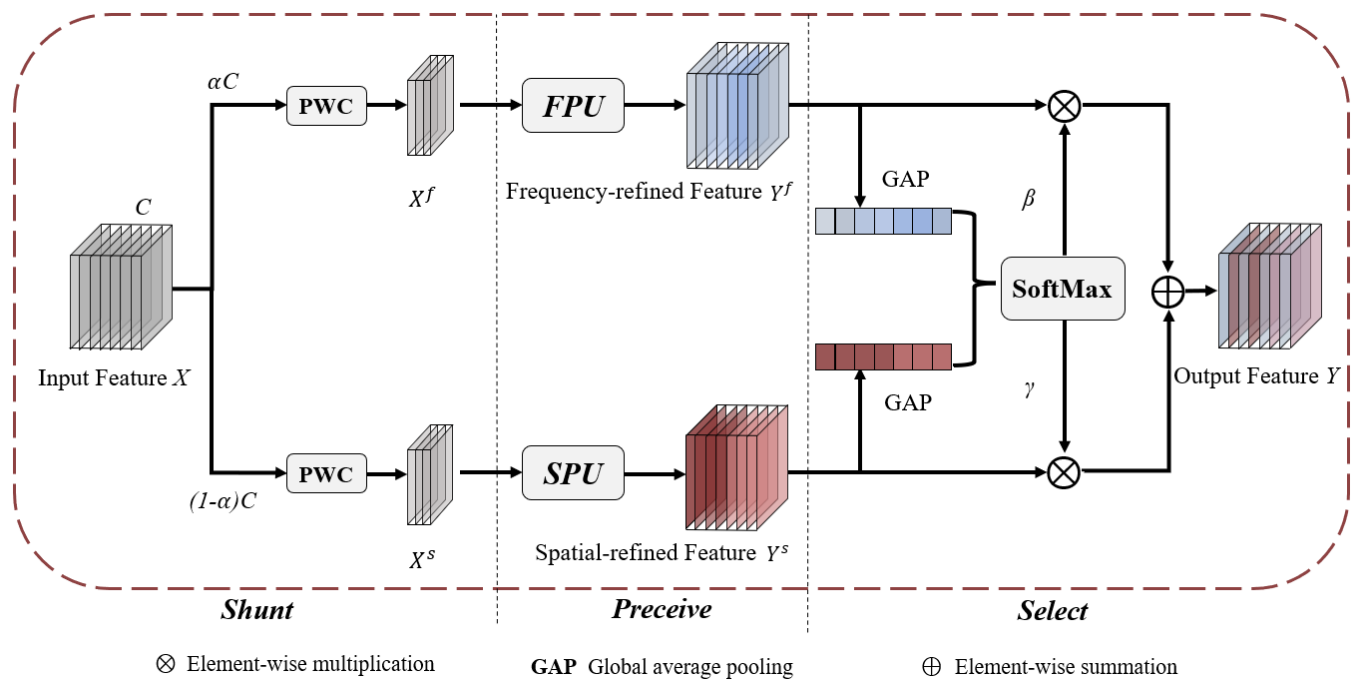
Network architecture of the SFSConv module. The module consists of three stages: Shunt, Perceive, and Select. Shunt: the input feature map *X* is split along channels into a spatial branch ((1−α)C) and a frequency branch (αC), each processed by 1 × 1 pointwise convolutions (PWC). Perceive: the spatial branch is refined via the Spatial Perception Unit (SPU) to obtain $Y^{s}$, and the frequency branch is refined via the Frequency Perception Unit (FPU) to obtain $Y^{f}$. Select: the outputs $Y^{s}$ and $Y^{f}$ are adaptively fused via global average pooling (GAP) and SoftMax to produce the final output feature *Y*.

In complex environments, single-domain spatial or frequency features often fail to fully represent the target. To address this, this study introduces the Spatial–Frequency Selective Convolution module (SFSConv) [26], which possesses multi-scale and multi-domain feature modeling capabilities, enabling the simultaneous capture of the spatial distribution, morphological structure, and texture features of figs. The SFSConv module consists of three units, namely the Spatial Perception Unit (SPU), the Frequency Perception Unit (FPU), and the Channel Selection Unit (CSU), as shown in Figure 6. The input features are first split into the spatial and frequency domains, which are then fed into the SPU and FPU, respectively, to finely model the fig’s location, shape, and texture features in complex environments. The SPU adapts to contextual information under different background conditions through dynamically adjustable convolution kernels, thereby characterizing the spatial relationship between the fruit and surrounding branches and leaves. The core of the FPU is a fractional-order Gabor transform, which is used to extract multi-scale, multi-directional high-frequency texture features, reducing background interference. Subsequently, the CSU adaptively selects and fuses the outputs from the SPU and FPU in a parameter-free manner, retaining the most representative and discriminative features.

The SFSConv module first divides the input feature map $X\in R^{C\times H\times W}$ into spatial and frequency components by a scaling factor α. The spatial part, denoted as $X^{s}\in R^{(1-\alpha )C\times H\times W}$, represents spatial features and provides spatial information. The frequency part, denoted as $X^{f}\in R^{\alpha C\times H\times W}$, captures frequency characteristics. Next, two separate 1×1 pointwise convolutions (PWCs) are applied to $X^{s}$ and $X^{f}$ to adjust the features, enhancing their compatibility for subsequent feature extraction in the spatial and frequency domains.

After feature adjustment, the spatial refinement feature $Y^{s}$ and the frequency refinement feature $Y^{f}$ ($Y^{s},Y^{f}\in R^{C\times H\times W}$), are extracted via the SPU and FPU modules, respectively, where *C* denotes the number of channels, and *H* and *W* are the height and width of the feature map. Subsequently, the CSU module adaptively fuses the two types of features, generating channel-wise statistical information $S^{s},S^{f}\in R^{C}$ through global average pooling (GAP), performing spatial compression for each channel.

(1) $$S^{n}=\mathrm{GAP}\left(Y^{n}\right)=\frac{1}{H\times W}\sum_{i=0}^{H-1}\sum_{j=0}^{W-1}Y_{i,j}^{\left(n\right)},\;\;n\in \left\{s,f\right\}$$

(2) $$\gamma =\frac{e^{S^{s}}}{e^{S^{s}}+e^{S^{f\;\;}}},\;\;\beta =\frac{e^{S^{f}}}{e^{S^{s}}+e^{S^{f}}}.$$

where $S^{s}$ and $S^{f}$ represent the channel-wise statistical information obtained from the spatial and frequency branches through global average pooling, while γ and β are the adaptive weights.

(3) $$Y=\gamma Y^{s}+\beta Y^{f}$$

where *Y* represents the final output feature.

#### 2.2.4. EBAM

In the field of computer vision, attention mechanisms have been widely applied to object detection [27]. The Bottleneck Attention Module (BAM) [28] is an efficient attention module designed to enhance the representational power of deep neural networks. Its uniqueness lies in its ability to infer attention maps through independent channels and spatial paths, strategically placed at the “bottleneck” of feature maps within the model, thereby constructing hierarchical attention.

**Figure 7:**
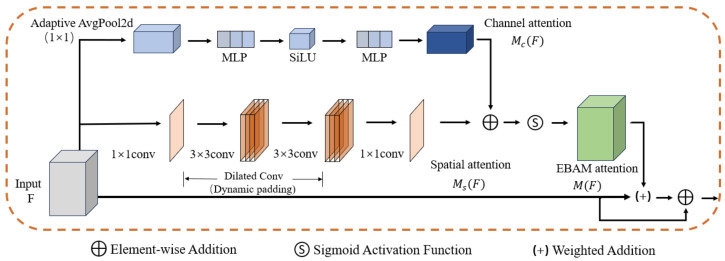
Network architecture of the EBAM module. The module has two branches: (1) a channel attention branch, consisting of Adaptive AvgPool2d, a multi-layer perceptron (MLP), and SiLU activation, which captures inter-channel dependencies and generates $M_{c}\left(F\right)$; (2) a spatial attention branch, using a bottleneck structure with dilated convolutions to capture contextual information and generate $M_{s}\left(F\right)$. The outputs of both branches are fused via addition and a Sigmoid function to produce the final attention map M(F), which is then weighted and combined with the input features for feature refinement.

To enhance the model’s detection capability for fig recognition tasks, this study designs an improved dual-branch attention module, EBAM, as shown in Figure 7. This module optimizes BAM, making it more effective at extracting key features of figs and suppressing occlusion interference. In terms of channel attention, EBAM replaces the traditional ReLU activation with SiLU, introducing a smoother nonlinear mapping that improves sensitivity to subtle differences in fruits. Additionally, the single-layer fully connected structure is expanded into a multi-layer perceptron (MLP), providing more flexibility in modeling channel dependencies. To avoid instability during small-batch training, the BatchNorm layer is removed, ensuring the robustness of the training process. In terms of spatial attention, EBAM introduces dynamic padding and configurable dilated convolutions, enabling the model to capture a larger receptive field and strengthen context modeling capabilities, addressing issues like branch and leaf occlusion. Furthermore, the channel branch adopts adaptive pooling instead of global average pooling with a fixed kernel size, eliminating dependence on the input feature map size and ensuring consistent multi-scale feature compression.

The EBAM module consists of two branches: the channel attention branch and the spatial attention branch. The channel branch models inter-channel dependencies through global average pooling and a multi-layer perceptron, while the spatial branch utilizes a bottleneck structure combined with dilated convolutions to capture a large receptive field and critical contextual location information. The outputs of both branches are unified in dimension, summed, and passed through a Sigmoid function to generate a 3D attention map, which is then weighted and combined with the input features to highlight critical channel and spatial information.

#### 2.2.5. MFCV

**Figure 8:**
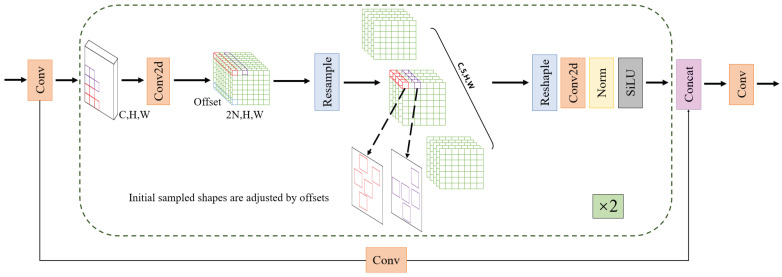
Network architecture of the MFCV module. The dashed box illustrates the core workflow of the module, including the dynamic sampling process implemented through offset-based resampling, as well as the multi-branch feature extraction structure composed of Conv2d, normalization, SiLU activation, and feature concatenation. The “×2” indicates that the structure within the dashed box is repeated twice.

To enhance the model’s performance in detecting small objects in complex natural environments, this study designs a Multi-Branch Dynamic Convolution Module (MFCV), as shown in Figure 8. This module combines the advantages of dynamic sampling convolution and the multi-branch residual feature fusion structure (C3k2), as illustrated in Figure 3.

In terms of structure, the MFCV first applies dynamic sampling convolution to the input features, predicting the offset (denoted as Offset) via a convolution operation, with dimensions of (B, 2N, H, W), where *B* is the batch size, and *N* is the number of sampling points for the convolution kernel. For example, with N=5, the offset is added to the original coordinates ($X_{0}+X_{n}$), resulting in corrected coordinates. Interpolation and resampling are then used to obtain the feature representations at the corresponding locations. This mechanism enables the model to flexibly handle spatial feature shifts caused by fruit size differences, irregular shapes, and lighting changes, significantly enhancing its perception of small objects and edge regions.

Subsequently, the feature map is passed through a multi-branch residual structure for deep feature extraction. MFCV inherits the multi-branch design philosophy of the C3k2 module, dividing the convolution output channels into multiple sub-branches, each containing a Bottleneck structure to capture local texture and global semantic features at different scales. The branches are efficiently fused through residual connections, enabling stable gradient propagation and improving the expression of small object features and the reconstruction of details.

Finally, the outputs from each branch are concatenated along the channel dimension, forming a fused feature map that combines sensitivity to local details and the ability to perceive global structure. Thanks to the synergistic effect of dynamic sampling and multi-branch fusion, MFCV demonstrates stronger feature resolution and localization accuracy in small fruit detection tasks.

(4) $$Conv\left(X_{0}\right)=\sum W\times \left(X_{0}+X_{n}\right)$$

The convolution operation under the dynamic sampling mechanism proceeds as follows. For each convolution sampling position $X_{0}$, the module first predicts the offset $X_{n}$ based on the network’s output, generating the dynamic sampling point $\left(X_{0}+X_{n}\right)$. Then, the weights of the convolution kernel *W* are applied to the offset sampling points. The convolution results from all sampling positions are summed, producing the output feature for that position.

### 2.3. Loss Function

The loss function is a core metric in deep learning, used to measure the difference between the model’s predictions and the true labels. It quantifies the model’s error and serves as the optimization objective, with algorithms such as gradient descent minimizing the loss to improve the model’s predictive ability.

In YOLOv11, the loss function jointly optimizes three core sub-tasks of object detection: bounding box localization, object classification, and confidence prediction. The total loss is the weighted sum of three components. For bounding box localization, CIoU Loss is used, which takes into account the intersection-over-union (IoU) between the predicted and ground truth boxes, the center point distance, and the aspect ratio, thereby improving the accuracy of bounding box predictions. Both object classification loss and confidence loss use cross-entropy loss: the former optimizes the prediction of target classes for positive samples, while the latter is used to distinguish between valid object boxes and background boxes.

In this experiment, CIoU Loss was also employed as the bounding box localization loss to ensure precise detection of fig targets.

### 2.4. Model Evaluation Metrics

To comprehensively assess the performance of the Fig-YOLO model in fig detection tasks, several evaluation metrics were used in this study. Precision (P) reflects the proportion of true figs among the targets identified as figs by the model, serving as a measure of detection accuracy. Recall (R) indicates the proportion of actual figs correctly detected, reflecting the completeness of the detection. Mean average precision (mAP) is used as a comprehensive metric. mAP@0.5 represents the detection performance when the Intersection over Union (IoU) threshold is set to 0.5, while mAP@50:95 averages the performance over IoU thresholds ranging from 0.5 to 0.95, providing a stricter evaluation of the model’s performance under different matching conditions. The F1 score is the harmonic mean of precision and recall, providing an overall measure of the model’s performance in balancing accuracy and completeness. These metrics allow for a systematic evaluation of the detection effectiveness of the Fig-YOLO model. The formulas for each metric are as follows:

(5) $$\begin{array}{cc} P & =\frac{TP}{TP+FP}\times 100\%,R=\frac{TP}{TP+FN}\times 100\% \end{array}$$

(6) $$\begin{array}{cc} AP & =\int_{0}^{1}P\left(r\right)\;dr,mAP=\frac{1}{N}\sum_{i=1}^{N}AP,F1=\frac{2\cdot P\cdot R}{P+R} \end{array}$$

In this case, TP refers to the number of true positive samples correctly predicted as positive, FP denotes the number of negative samples incorrectly predicted as positive, and FN represents the number of positive samples incorrectly predicted as negative. P(r) refers to the precision at the recall rate *r*. *N* denotes the number of target categories. The F1 score is the harmonic mean of precision and recall, providing a comprehensive measure of the model’s performance in predicting both positive and negative samples. Furthermore, this study adopts inference time (Inf.), frames per second (FPS), and computational cost (GFLOPs) as key indicators of real-time performance. Shorter inference time, higher FPS, and lower GFLOPs indicate reduced computational latency and improved operational efficiency of the model.

### 2.5. Experimental Platform and Parameter Settings

For the fig detection task, the Fig-YOLO model was developed and trained in the Pytorch 2.5.1 environment. The experimental platform configuration is as follows: the CPU is an Intel(R) Xeon(R) Platinum 8352V @ 2.10 GHz, the GPU is an NVIDIA GeForce RTX 4090 (24 GB VRAM), the operating system is Ubuntu 22.04, and the acceleration environment is CUDA 12.1. The programming environment includes Python 3.12 and OpenCV 4.6. The hyperparameters for the training phase are shown in Table 2. The training process involved YOLOv11’s built-in staged strategy, combined with a cosine annealing learning rate scheduler to maintain optimization stability and improve model accuracy in the later stages of training. Fig-YOLO was implemented by modifying the official YOLOv11 framework, and the source code and accompanying instructions can be provided to researchers upon reasonable request.

In terms of hardware evaluation, the experimental platform adopted in this study has good computational performance and accessibility under current research conditions. The NVIDIA RTX 4090 graphics card provides ample computing power for model training, enabling us to complete experiments and conduct necessary model iterations within a reasonable time frame. This configuration is not an extremely high-end device, but a commonly used research platform that balances performance and cost, with good representativeness and reproducibility. It should be emphasized that there are differences in hardware requirements between the model training phase and the actual deployment environment. In this study, the inference time and FPS of the model are reported to reflect the efficiency performance that may be encountered in practical applications. These metrics can provide a reference benchmark for subsequent migration and deployment on different hardware platforms.

## 3. Results and Discussion

### 3.1. Ablation Experiment

To evaluate the effectiveness of the three improvements, namely the EBAM, SFSConv, and MFCV modules, ablation experiments were conducted based on the YOLOv11 model. All experiments in this study were conducted without loading any pre-trained weights, and the training parameters were kept consistent across models to ensure a fair comparison of the improvement effects of different methods. The experimental results are shown in Table 3.

By progressively introducing the EBAM, SFSConv, and MFCV modules, the performance of the YOLOv11 model in the fig detection task was significantly improved. Compared to the baseline model, after introducing the EBAM module, P, R, mAP@0.5, and F1 score were increased to 87.7%, 77.2%, 85.6%, and 82.2%, respectively. This improvement is mainly attributed to the module’s ability to enhance the model’s attention to key features, making feature extraction more accurate. However, the individual introduction of the SFSConv or MFCV module also brought varying degrees of performance improvement. Among them, SFSConv showed the most significant improvement in precision, while MFCV had a more notable effect on improving recall. When both EBAM and SFSConv were introduced together, the model’s performance was further enhanced, with P increasing to 89.6%, R to 78.0%, and F1 reaching 83.5%. This demonstrated the clear synergistic effect of the two modules in improving the model’s overall recognition ability. Similarly, the combination of EBAM and MFCV improved recall and F1 score, while the union of SFSConv and MFCV also led to an overall performance boost. Ultimately, when all three modules, EBAM, SFSConv, and MFCV, were introduced simultaneously, the model achieved optimal performance across all evaluation metrics. P increased to 89.2%, R to 78.4%, mAP@0.5 to 87.3%, mAP@50:95 to 67.4%, and F1 reached 83.6%. In conclusion, the synergistic effect of the three modules significantly enhanced the overall performance of the YOLOv11 model in the fig detection task.

#### 3.1.1. Comparison and Analysis of Typical Attention Mechanisms

To validate the effectiveness of the EBAM module in the fig detection task, comparative experiments were conducted with several typical attention mechanisms, as shown in Table 4. The experimental results indicate that EBAM achieves a P of 87.7%, R of 77.2%, mAP@0.5 of 85.6%, and mAP@50:95 of 64.6%. Although CBAM attains a slightly higher P of 89.8%, its R drops to 74.2%, suggesting that EBAM is more effective in detecting small and occluded fig fruits. Compared with SimAm and C2BRA, EBAM improves mAP@0.5 by 0.9–1.6%, demonstrating a clear advantage in overall detection accuracy. Furthermore, EBAM maintains a balanced performance between P and R, achieving the highest F1 of 82.2%, reflecting its robustness and stability across different IoU thresholds. These results quantitatively confirm that EBAM provides a more reliable attention mechanism for fig detection under complex field conditions.

#### 3.1.2. Ablation Experiment of Different Convolution Modules and Attention Modules

The experimental results are shown in Table 5. Introducing different convolution modules based on the EBAM attention module has a significant impact on fig detection performance. The combination of EBAM and SFSConv achieves the best performance across all key metrics, with a P of 89.6%, R of 78.0%, mAP@0.5 of 87.0%, mAP@50:95 reaching 66.5%, and F1 of 83.5%. In contrast, while the combination of EBAM and GSConv achieves a slightly higher P of 88.4%, its R drops to 74.3%, indicating that the lightweight GSConv has limitations in extracting features of small fig fruits. Similarly, EBAM + GhostConv and EBAM + SPDConv exhibit inferior overall performance, with F1 scores of 81.9% and 82.2%, respectively. GhostConv, as a lightweight network design, has limited feature representation capacity, while SPDConv, although designed for small targets, fails to fully exploit the spatial-frequency information relevant to fig detection. Compared with these alternative modules, EBAM+SFSConv improves mAP@0.5 by 2.0–3.0% and F1 by 1.3–2.5%, quantitatively demonstrating a better balance between precision and recall. These results confirm that SFSConv effectively complements EBAM, providing a robust and efficient convolution–attention combination for fig detection under complex conditions.

#### 3.1.3. Comparison and Analysis of Feature Fusion Modules

**Figure 9:**
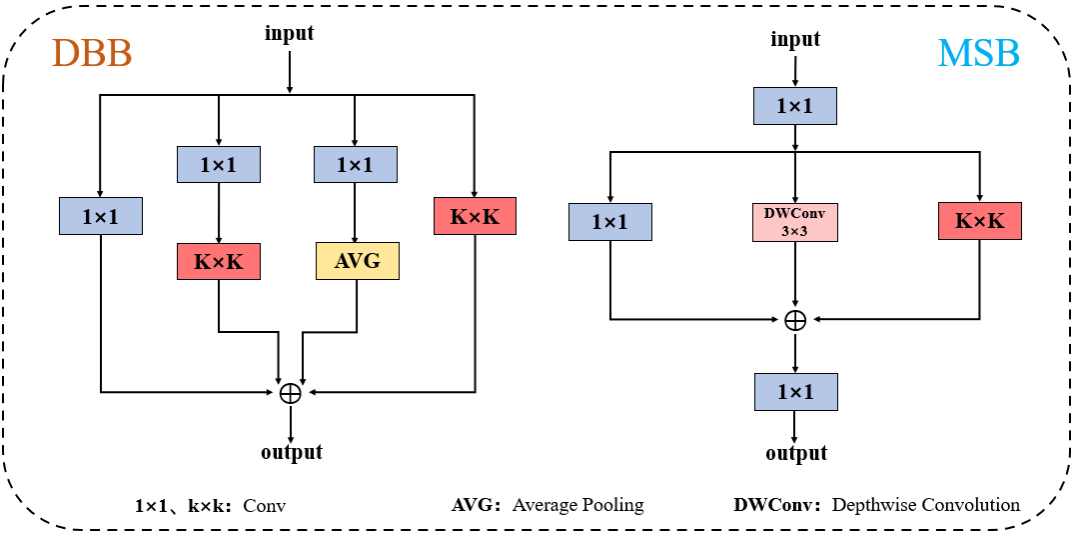
Structure of DiverseBranchBlock and MSBlock. “⊕” denotes residual connection.

As shown in Table 6, the proposed MFCV module achieves a P of 88.8%, R of 76.7%, mAP@0.5 of 85.6%, mAP@50:95 of 64.5%, F1 of 82.5%, and a computational cost of 7.2 GFLOPs. Compared with C3k2_MSBlock, MFCV improves P, R, mAP@0.5, and F1 by 0.7%, 1.0%, 0.7%, and 0.8%, respectively, demonstrating robust performance despite a slightly higher computational cost (7.2 compared with 5.9 GFLOPs). In comparison with DiverseBranchBlock, MFCV shows a slightly higher P (by 0.2%) and a slightly lower R (by 0.2%), while mAP@0.5 and F1 are comparable, yet it maintains lower computational overhead (7.2 compared with 8.9 GFLOPs), indicating more efficient feature fusion. The performance gains are mainly attributed to MFCV’s ability to adaptively integrate shallow and deep features, allowing flexible modeling of fruit size and shape variations. In contrast, C3k2_MSBlock is limited by its fixed convolution kernels in handling different fruit scales, and although DiverseBranchBlock enhances feature representation through multiple branches, each branch requires additional computation and fusion, increasing training and inference costs (Figure 9). Overall, MFCV provides an efficient and robust feature modeling and fusion solution for fig detection under complex scenarios.

### 3.2. Comparative Experiment

To evaluate the performance of the Fig-YOLO model in the fig detection task, we compared it with various existing object detection models, including Faster R-CNN [29], SSD [30], YOLOv5 [31], YOLOv6 [32], YOLOv8 [33], YOLOv10 [34], and YOLOv11. The experimental results are shown in Table 7.

**Figure 10:**
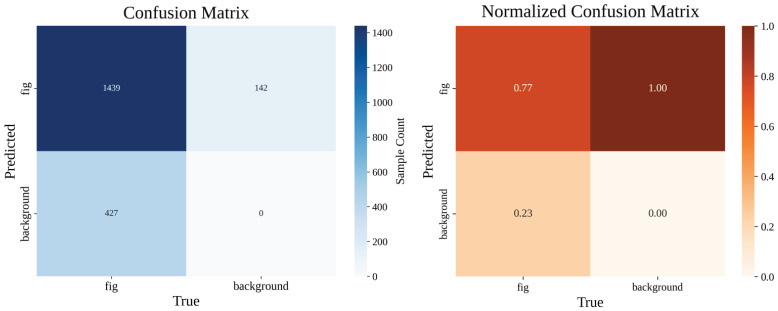
Confusion matrix and normalized confusion matrix of Fig-YOLO on the test dataset.

Fig-YOLO demonstrates a clear advantage across key metrics, with a P of 89.2%, R of 78.4%, mAP@0.5 of 87.3%, and an F1 of 83.6%, significantly outperforming the other models. The model’s confusion matrix is shown in Figure 10. The left panel presents the confusion matrix, illustrating the distribution of predicted sample counts for figs and background in the test set, while the right panel shows the normalized confusion matrix, representing the prediction probabilities for each class. Compared to YOLOv11, Fig-YOLO shows improvements in both precision and recall, indicating its stronger detection capability in complex backgrounds and dense fruit environments. While YOLOv10 and YOLOv8 perform well in mAP@0.5, they still fall short of Fig-YOLO in precision, recall, and F1 score.

Additionally, Fig-YOLO introduces spatial–frequency convolution modules, attention mechanisms, and feature fusion modules, demonstrating strong detection capability while maintaining high precision. Compared to the traditional SSD, Fig-YOLO performs better in both precision and recall. In comparison to Faster R-CNN, Fig-YOLO not only maintains high precision and recall but also achieves significant improvements in F1 score and mAP, showcasing its comprehensive advantages in fig detection tasks. In terms of real-time performance, YOLOv10 exhibits the shortest inference time (1.6 ms) and the highest frame rate (625 FPS), demonstrating a clear advantage in speed, while YOLOv8 and YOLOv5 also achieve relatively fast inference. However, despite their high computational efficiency, these lightweight models still underperform Fig-YOLO in terms of accuracy metrics. In comparison, Fig-YOLO has an inference time of 6.4 ms and a frame rate of 156.3 FPS. Although slightly slower than some lightweight models, it still satisfies real-time detection requirements and maintains superior performance across all accuracy metrics, demonstrating a well-balanced trade-off between speed and precision.

**Figure 11:**
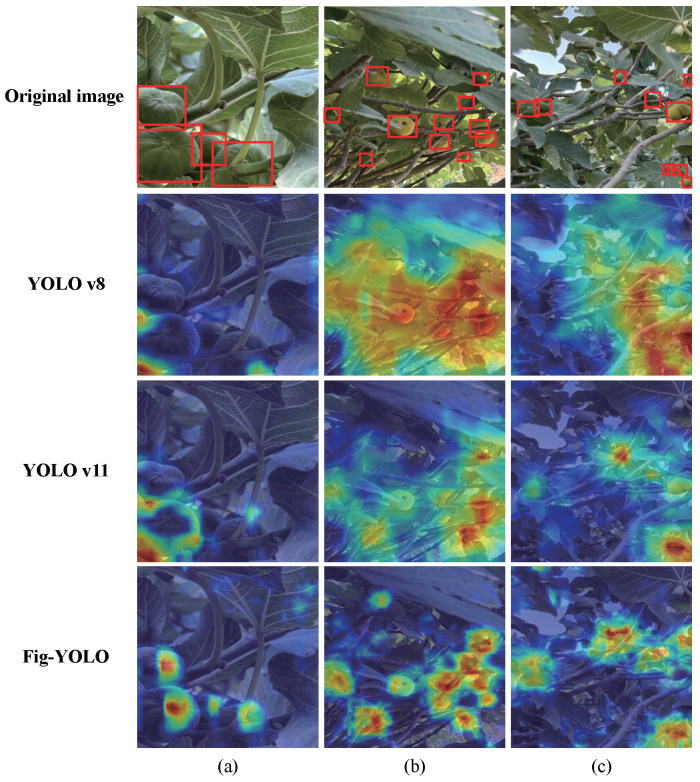
Comparison of heatmaps before and after model optimization. Red rectangles indicate fig fruits in the original images. Note: (**a**) The image depicts a scenario with concentrated fruit distribution; (**b**) the image shows a scenario with dispersed and partially occluded fruit; (**c**) the image illustrates a scenario with fruit in a complex background.

To intuitively present the differences before and after model optimization, this study uses Grad-CAM to perform a visual analysis of the feature responses of YOLOv8, YOLOv11, and Fig-YOLO, as shown in Figure 11. In the three scenarios (a), (b), and (c), the heatmaps of Fig-YOLO consistently perform better than those of YOLOv11 and YOLOv8. In Figure 11a, the YOLOv8 heatmap does not accurately cover the fruit region, while the YOLOv11 heatmap also has limited focus on the fruit, with false activations in non-fruit areas. In contrast, the Fig-YOLO heatmap provides a more precise and robust coverage of the fruit region, clearly highlighting the location and shape of the fruit. In Figure 11b, the YOLOv8 heatmap is very scattered and struggles to capture features of dispersed and occluded fruits, while the YOLOv11 heatmap exhibits similar issues. In comparison, the Fig-YOLO heatmap is denser and can accurately focus on the fruit region, even when the fruit is occluded or unevenly distributed, allowing for better identification and feature emphasis. In Figure 11c, the YOLOv8 heatmap shows weak attention to the fruit in a complex background, making it difficult to clearly distinguish the fruit’s position. The YOLOv11 heatmap also misses some fruit in the complex background. However, the Fig-YOLO heatmap effectively suppresses background interference, concentrating the attention on the fruit and clearly displaying its distribution. Overall, Fig-YOLO can more accurately focus on the fig fruit region in various scenarios.

### 3.3. Analysis of Data Augmentation Effectiveness

**Figure 12:**
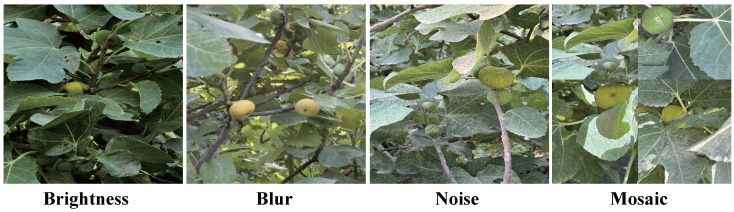
Partial data augmentation samples.

The data augmentation strategies employed in this study include flipping, rotation, brightness adjustment, blurring, noise addition, and mosaic processing, as shown in Figure 12. Through these operations, the model is able to learn fruit features under different directions, brightness levels, and local disturbances during the training phase.

To evaluate the impact of data augmentation on the performance of the Fig-YOLO model, this study compared the training results with and without data augmentation: The experimental results are shown in Table 8. After introducing data augmentation, the model showed improvements across all performance metrics: P and R increased from 87.4% and 74.9% to 89.2% and 78.4%, respectively; mAP@0.5 increased to 87.3%; mAP@50:95 improved to 67.4%; and the F1 score rose to 83.6%. These results indicate that data augmentation significantly enhances the model’s ability to adapt to diverse fruit features, thereby improving overall detection accuracy.

**Figure 13:**
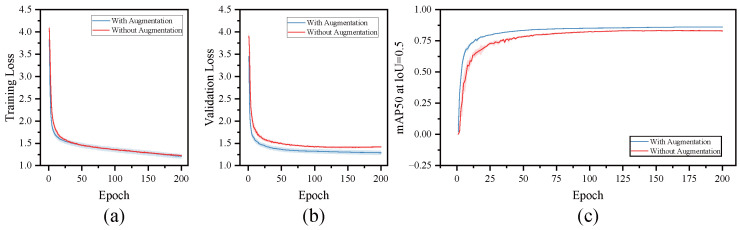
Comparison of training results with and without data augmentation. (**a**) Comparison of training loss changes with and without data augmentation. (**b**) Comparison of validation loss changes with and without data augmentation. (**c**) Comparison of mAP@0.5 performance with and without data augmentation at IoU = 0.5.

To further analyze the impact of the data augmentation strategy on the model’s training process, this study plotted the training loss, validation loss, and mAP@0.5 curves, with shaded areas representing the variability across independent runs using different random seeds, as shown in Figure 13. From the figure, it can be seen that after applying data augmentation, both the training loss (Figure 13a) and validation loss (Figure 13b) decrease more rapidly and steadily, indicating that the augmentation strategy effectively mitigates overfitting and accelerates training convergence. Meanwhile, the mAP@0.5 curve (Figure 13c) overall performs better, validating the positive effect of enhanced data diversity on the model’s detection performance.

### 3.4. Feasibility Analysis of Fig-YOLO in Complex Environments

To validate the application potential of the Fig-YOLO model in real-world scenarios, we conducted a comprehensive analysis of its feasibility in complex environments. Fig fruits exhibit size variations, and the lighting conditions and occlusion levels in orchard environments can affect detection accuracy. Therefore, based on the 2025 test set, this study introduced independent supplementary samples collected from the Azihan Fig Ecological Park in 2024 for external validation. These images were not used in model training. The two-year datasets differ significantly in terms of collection time, location, equipment, and image resolution, providing an evaluation of Fig-YOLO’s performance across year-to-year and scene-to-scene data, as shown in Table 1. To further evaluate the model, this study incorporates fig images from publicly available international datasets. These images cover different varieties, acquisition conditions, and background environments, enabling an assessment of Fig-YOLO’s performance across regions and data sources. A systematic analysis of detection results under varying fruit sizes, occlusion levels, lighting conditions, and data sources facilitates a comprehensive evaluation of the model’s stability and adaptability in complex environments.

#### 3.4.1. Different Size Scenarios

**Figure 14:**
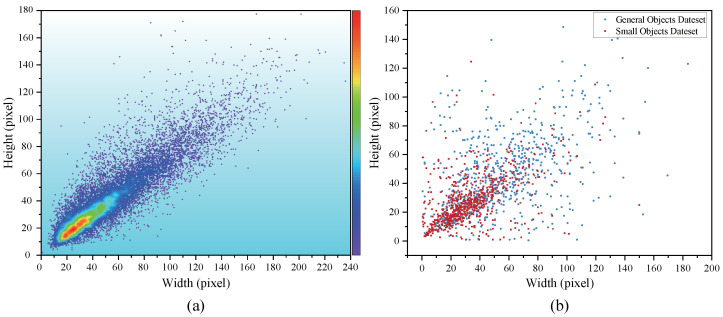
Comparison of Pixel distribution for objects of different sizes: (**a**) Overall dataset. (**b**) Test subset.

The dominance of small objects in the dataset highlights the importance of testing different object sizes in practical scenarios. As shown in Figure 14, Figure 14a presents the pixel distribution of target width and height in the dataset, with color intensity indicating the target density. It is evident that small objects occupy a larger proportion and are concentrated within smaller pixel ranges. Figure 14b shows the target size distribution of two test subsets, where red points represent the small object dataset (with a higher proportion), with their target width and height mainly located in lower pixel ranges; blue points represent the general object dataset, exhibiting a wider distribution of sizes. The x-axis and y-axis in the figure both represent 640 pixels.

There are significant size differences in the fruits, which directly affect the accuracy of the object detection model. As shown in Table 9, in small target detection, P, R, and mAP@0.5 are 85.7%, 74.8%, and 82.5%, respectively, which is significantly lower than for general objects, where P is 92.3%, R is 82.6%, and mAP@0.5 is 91.5%. Additionally, the mAP@50:95 for small targets is 62.2%, much lower than the 71.9% for general objects, and the F1 score is also lower, with 79.9% for small targets compared to 87.2% for general objects.

**Figure 15:**
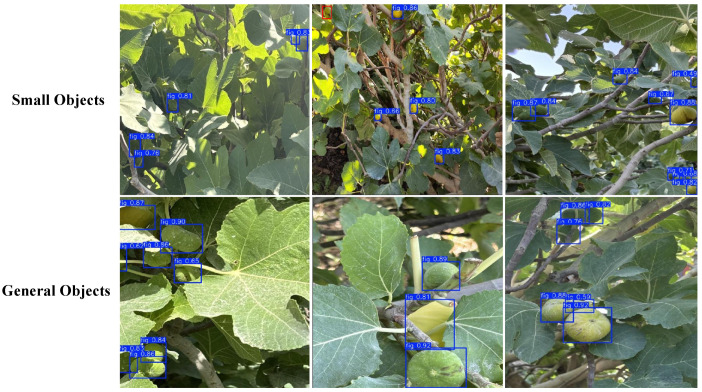
Model recognition under different size conditions. The fruits marked with red boxes are false negatives.

As shown in Figure 15, one possible reason for this discrepancy is that small targets, due to their limited pixel coverage, may lose boundary and texture features during downsampling and feature extraction, resulting in insufficient feature representation and ultimately affecting detection performance. In contrast, general objects have a higher pixel coverage in the image, allowing the model to capture more complete geometric and texture information, leading to better detection performance.

#### 3.4.2. Different Occlusion Conditions

**Figure 16:**
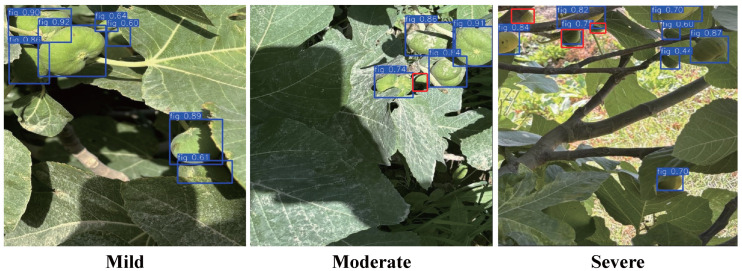
Model recognition under different occlusion conditions. The fruits marked with red boxes are false negatives.

Occlusion is one of the main challenges faced during fig detection, particularly in orchard environments where fruit overlap and branch or leaf cover are common. These factors significantly impact detection accuracy and stability. To comprehensively evaluate the performance of Fig-YOLO under different occlusion conditions, the test set was divided into three categories: light occlusion (≤30%), moderate occlusion (30–70%), and heavy occlusion (≥70%). The experimental results are shown in Table 10 and Figure 16.

Under different occlusion conditions, the model’s recognition results align with the performance data. In the mild occlusion, the model performed best, with a P of 91.0%, R of 80.2%, mAP@0.5 of 87.8%, and an F1 of 85.3%. The majority of the fruits were accurately detected, with high confidence scores and nearly complete detection. As the occlusion degree increased, the performance slightly declined. Under moderate occlusion, the recall dropped to 79.1%, but Fig-YOLO still maintained good detection performance (mAP@0.5 of 87.0%). In the case of heavy occlusion, the model performance decreased most significantly, with mAP@0.5 dropping to 78.9%. Some heavily occluded fig fruits were not detected by the model (indicated by red boxes). The figure shows that these fruits grow in clusters and are extensively occluded by leaves and branches. This visual example demonstrates that severe occlusion leads to the loss of fruit boundaries and texture features, thereby reducing the model’s ability to correctly identify these fruits and resulting in missed detections. In contrast, for partially visible or lightly occluded fruits (indicated by blue boxes), the model is still able to detect them successfully, indicating relatively stable recognition performance under complex occlusion conditions. This visual analysis clearly illustrates the limitations of the model under extreme occlusion scenarios.

#### 3.4.3. Performance Under Different Lighting Conditions

**Figure 17:**
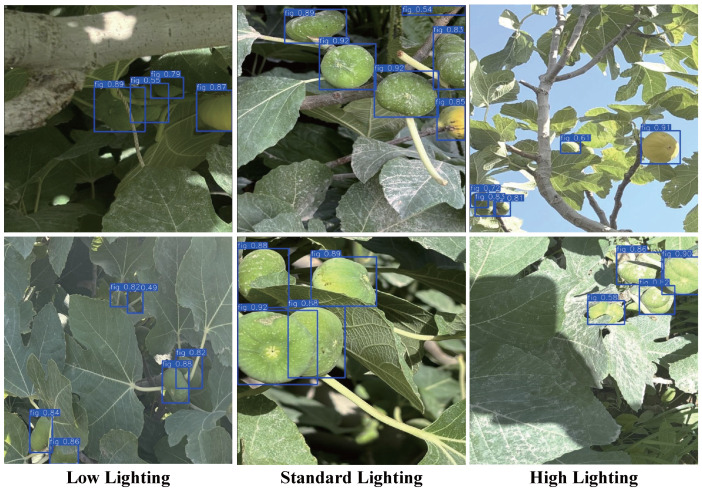
Model recognition under different lighting conditions.

This study further evaluates the performance of the Fig-YOLO model under various lighting conditions, including standard lighting (daylight), low light (simulating dusk/cloudy scenes), and high light (direct strong sunlight). As shown in Figure 17, under standard lighting, the model achieved the best performance, accurately detecting the fruits with high confidence. The data in Table 11 further confirm this, with an mAP@0.5 of 90.2%, P of 89.6%, and R of 83.4%, showing stable overall performance. Under high light conditions, the accuracy slightly decreased due to surface reflection and glare interference, with mAP@0.5 dropping to 85.3%. In low light conditions, image contrast was reduced, and fruit visibility decreased, leading to a “pseudo-occlusion” phenomenon. Although the model could still detect the fruits, the confidence of some detections significantly dropped (the lowest being 0.49), and the overall mAP@0.5 decreased to 81.6%. Despite these challenges, thanks to Fig-YOLO’s improved convolution modules and attention mechanism, the model maintained relatively high accuracy and stability across all three lighting conditions.

#### 3.4.4. Validation of the Model on Samples from Different Sources

To further evaluate the model’s adaptability across different regions and varieties, two different types of external samples were selected for validation: images of fig fruits from other regions and varieties (Sample 1, from the Roboflow fig-fruit_dataset) and images of a different fruit type, citrus (Sample 2, from the Roboflow citrus-kepw7_dataset). The validation results are summarized in Table 12. For Sample 1, the model achieved a precision of 86.6% and an mAP@0.5 of 80.3%, slightly lower than that on the original validation set. Although the predicted bounding boxes generally exhibited high confidence scores, the mAP@0.5 was somewhat reduced, primarily due to factors such as annotation boxes covering the fruit diameter and relatively large bounding boxes, which introduced variations in target scale and shape for the regression task. Nevertheless, the model maintained acceptable detection performance on Sample 1.

**Figure 18:**
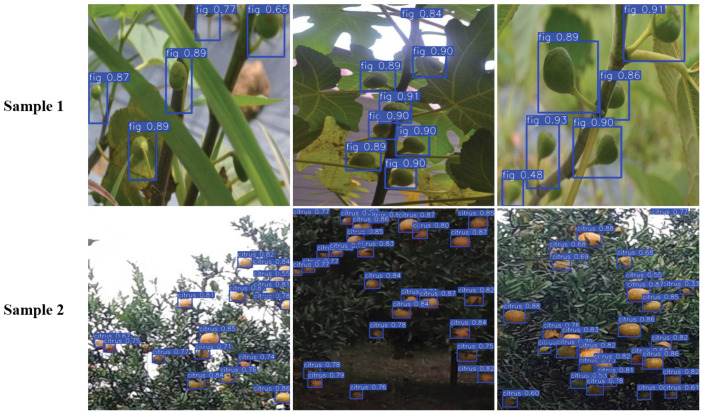
Detection results of the model on two different datasets.

In contrast, Sample 2 contained fruits with more distinct boundaries and color features, enabling the model to learn more stable and transferable feature representations. Consequently, the model achieved a precision of 90.9%, an mAP@0.5 of 94.1%, and an F1 score of 89.2% on this sample, indicating that consistent annotation practices and prominent fruit characteristics substantially enhance the model’s performance across different fruit types. Figure 18 illustrates the detection results on the different external samples. Overall, the model demonstrates robust detection capability across two datasets.

## 4. Conclusions

This study proposes an image-based fig detection method for modern agriculture, shifting from traditional manual observation and classical image processing techniques to deep learning-based automatic recognition, thereby enhancing the accuracy of fruit detection. Based on the YOLOv11n architecture, we designed the Fig-YOLO model and introduced the SFSConv, EBAM, and MFCV modules to enhance the model’s adaptability to fig fruit features, occlusion, and lighting variations in complex environments. Specifically, the SFSConv module improves fruit boundary segmentation accuracy and enhances the ability to differentiate against texture-similar backgrounds by jointly modeling spatial and frequency domain features with spatial perception and frequency perception units. The EBAM module employs a channel and spatial dual-branch attention mechanism to effectively highlight key fruit features and suppress interference from complex backgrounds and occlusions. The MFCV module adapts to capture the features of figs in various sizes and shapes through dynamic sampling and residual deep feature fusion, improving small object detection accuracy. The ablation experiment results show that the Fig-YOLO model, after integrating the three modules, performs excellently in the fig detection task. The precision (P), recall (R), mAP@0.5, mAP@50:95%, and F1 score reach 89.2%, 78.4%, 87.3%, 67.4%, and 83.6%, respectively, significantly outperforming the models using only a single module or the unmodified YOLOv11n. Additionally, we validated the model’s feasibility in complex environments, with the results indicating that, compared to small targets and lighting variations, severe occlusion conditions have a more significant impact on detection performance.

Future research can further explore performance optimization strategies for severe occlusion conditions to improve the robustness of fruit detection in complex environments. The model can also be extended to fruit maturity detection tasks, enabling precise identification and grading of fruit maturity status, providing technological support for intelligent harvesting and quality assessment.

## Figures and Tables

**Table 1:** Overview of the fig image dataset.

Time	Location	Images	Device	Resolution	Usage
October 2024	Eco-park	950	Redmi Note12 Turbo	3472 × 4624	Supplementary Test
July 2025	Jinjiamu Orchard	1470	iPhone 15 Plus	4284 × 5712	Train/Validation/Test

**Table 2:** Hyperparameter settings.

Item	Value
Input image size	640×640
Batch size	16
Initial learning rate	0.01
Weight decay	0.0005
Momentum	0.937
Optimizer	SGD
Workers	4
Epochs	200

**Table 3:** Ablation experiment results. ✓: The module is added in the model; ×: The module is not added.

Model	EBAM	SFSConv	MFCV	P	R	mAP@0.5	mAP@50:95	F1
				(%)	(%)	(%)	(%)	(%)
YOLOv11 (Baseline)	×	×	×	86.8	77.0	85.3	64.5	81.6
+EBAM	✓	×	×	87.7	77.2	85.6	64.6	82.2
+SFSConv	×	✓	×	89.3	77.5	86.4	66.6	83.2
+MFCV	×	×	✓	88.8	76.7	85.6	64.5	82.4
EBAM + SFSConv	✓	✓	×	89.6	78.0	87.0	66.5	83.5
EBAM + MFCV	✓	×	✓	88.5	76.0	85.5	64.4	81.9
SFSConv + MFCV	×	✓	✓	88.4	78.1	87.1	67.0	83.0
EBAM + SFSConv + MFCV (Ours)	✓	✓	✓	89.2	78.4	87.3	67.4	83.6

**Table 4:** Comparison analysis of EBAM and typical attention mechanisms. Note: Bold = best, * = worst.

Module	P	R	mAP@0.5	mAP@50:95	F1
	(%)	(%)	(%)	(%)	(%)
-	86.8	77.0	85.3	64.5	81.6
C2BRA	87.1	77.4	85.0	64.2	82.2
SimAm *	88.6	75.8	84.7	64.0	81.9
SE	86.0	77.0	85.1	64.1	81.3
CBAM	89.8	74.2	85.4	64.4	81.5
EBAM (Ours)	**87.7**	**77.2**	**85.6**	**64.6**	**82.2**

**Table 5:** Ablation experiment of different convolution modules and attention modules. Note: Bold = best, * = worst.

Module	P	R	mAP@0.5	mAP@50:95	F1
	(%)	(%)	(%)	(%)	(%)
EBAM	87.7	77.2	85.6	64.6	82.2
EBAM + GSConv *	88.4	74.3	84.0	63.6	80.9
EBAM + GhostConv	87.2	77.0	85.0	63.8	81.9
EBAM + SPDConv	88.5	76.4	85.1	63.8	82.2
EBAM + SFSConv (Ours)	**89.6**	**78.0**	**87.0**	**66.5**	**83.5**

**Table 6:** Comparison between MFCV and representative feature fusion modules. Note: Bold = best, * = worst.

Module	P	R	mAP@0.5	mAP@50:95	F1	GFLOPs
	(%)	(%)	(%)	(%)	(%)	
-	86.8	77.0	85.3	64.5	81.6	6.3
C3k2_MSBlock *	88.1	75.7	84.9	63.6	81.7	5.9
DiverseBranchBlock	88.6	76.9	85.5	64.5	82.5	8.9
MFCV (Ours)	**88.8**	**76.7**	**85.6**	**64.5**	**82.5**	**7.2**

**Table 7:** Experimental results of different models. Note: Bold = best, * = worst.

Model	P	R	mAP@0.5	mAP@50:95	F1	Inf.	FPS
	(%)	(%)	(%)	(%)	(%)	(ms)	
Faster R-CNN *	75.1	72.0	75.6	–	73.5	–	–
SSD	88.9	72.4	81.8	–	79.9	–	–
YOLOv5	88.8	74.4	84.5	63.5	80.8	2.7	370.0
YOLOv6	87.0	76.3	84.5	63.9	81.5	3.0	333.3
YOLOv8	87.2	77.0	84.8	63.9	81.9	2.6	384.6
YOLOv10	87.6	76.2	84.6	64.2	81.8	1.6	625.0
YOLOv11	86.8	77.0	85.3	64.5	81.6	2.8	357.0
Fig-YOLO (Ours)	**89.2**	**78.4**	**87.3**	**67.4**	**83.6**	**6.4**	**156.3**

**Table 8:** Model performance with and without data augmentation.

Augmentation Strategy	P	R	mAP@0.5	mAP@50:95	F1
	(%)	(%)	(%)	(%)	(%)
Without Augmentation	87.4	74.9	83.9	62.4	80.7
With Augmentation	89.2	78.4	87.3	67.4	83.6

**Table 9:** Detection performance under different size scenarios.

Size Level	P	R	mAP@0.5	mAP@50:95	F1
	(%)	(%)	(%)	(%)	(%)
Small Objects	85.7	74.8	82.5	62.2	79.9
General Objects	92.3	82.6	91.5	71.9	87.2

**Table 10:** Detection results under different occlusion conditions.

Occlusion Level	P	R	mAP@0.5	mAP@50:95	F1
	(%)	(%)	(%)	(%)	(%)
Mild Occlusion	91.0	80.2	87.8	72.4	85.3
Moderate Occlusion	86.4	79.1	87.0	65.4	82.6
Severe Occlusion	84.0	70.0	78.9	56.8	76.4

**Table 11:** Detection performance under different lighting conditions.

Lighting Conditions	P	R	mAP@0.5	mAP@50:95	F1
	(%)	(%)	(%)	(%)	(%)
Low Lighting	85.8	72.5	81.6	61.2	78.6
Standard Lighting	89.6	83.4	90.2	69.6	86.5
High Lighting	87.1	76.4	85.3	63.9	81.3

**Table 12:** Detection performance on external test samples.

Sample	P	R	mAP@0.5	F1
	(%)	(%)	(%)	(%)
Validation Set	89.2	78.4	87.3	83.6
Sample 1 (fig, different variety)	86.6	73.8	80.3	79.7
Sample 2 (citrus, different fruit type)	90.9	87.7	94.1	89.2

## Data Availability

The original contributions presented in the study are included in the article; further inquiries can be directed to the corresponding authors.
